# Group-level stability but individual variability of neurocognitive status after awake resections of right frontal IDH-mutated glioma

**DOI:** 10.1038/s41598-022-08702-2

**Published:** 2022-04-12

**Authors:** Marion Barberis, Isabelle Poisson, Valentine Facque, Sophie Letrange, Cécile Prevost-Tarabon, Emmanuel Houdart, Sébastien Froelich, Richard Levy, Emmanuel Mandonnet

**Affiliations:** 1 Department of Neurosurgery, Lariboisière Hospital, AP-HP, 2 rue Ambroise Paré, 75010 Paris, France; 2 Frontlab, CNRS UMR 7225, INSERM U1127, Paris Brain Institute (ICM), Paris, France; 3 Department of Neuroradiology, Lariboisière Hospital, AP-HP, Paris, France; 4 Department of Neurology, Pitié-Salpêtrière Hospital, AP-HP, Paris, France; 5 Université de Paris, Paris, France

**Keywords:** Surgical oncology, CNS cancer

## Abstract

Awake surgery for low-grade gliomas is currently considered the best procedure to improve the extent of resection and guarantee a "worth living life" for patients, meaning avoiding not only motor but also cognitive deficits. However, tumors located in the right hemisphere, especially in the right frontal lobe, are still rarely operated on in awake condition; one of the reasons possibly being that there is little information in the literature describing the rates and nature of long-lasting neuropsychological deficits following resection of right frontal glioma. To investigate long-term cognitive deficits after awake surgery in right frontal IDH-mutated glioma. We retrospectively analyzed a consecutive series of awake surgical resections between 2012 and 2020 for right frontal IDH-mutated glioma. We studied the patients' subjective complaints and objective neuropsychological evaluations, both before and after surgery. Our results were then put in perspective with the literature. Twenty surgical cases (including 5 cases of redo surgery) in eighteen patients (medium age: 42.5 [range 26–58]) were included in the study. The median preoperative volume was 37 cc; WHO grading was II, III and IV in 70%, 20%, and 10% of cases, respectively. Preoperatively, few patients had related subjective cognitive or behavioral impairment, while evaluations revealed mild deficits in 45% of cases, most often concerning executive functions, attention, working memory and speed processing. Immediate postoperative evaluations showed severe deficits of executive functions in 75% of cases but also attentional deficits (65%), spatial neglect (60%) and behavioral disturbances (apathy, aprosodia/amimia, emotional sensitivity, anosognosia). Four months after surgery, although psychometric z-scores were unchanged at the group level, individual evaluations showed a slight decrease of performance in 9/20 cases for at least one of the following domains: executive functions, speed processing, attention, semantic cognition, social cognition. Our results are generally consistent with those of the literature, confirming that the right frontal lobe is a highly eloquent area and suggesting the importance of operating these patients in awake conditions.

## Introduction

Over the past decade, numerous studies have provided cumulative evidence that the extent of resection is a strong predictor of prolonged survival in (IDH-mutated) diffuse low-grade glioma (DLGG) patients^1–4^. Importantly, the effect of surgery has been observed regardless of the IDH-mutated subtypes—1p19q-codeleted oligodendroglioma or 1p19q noncodeleted astrocytoma^5–7^. Accordingly, surgical resection of DLGG is now considered as the first option in the guidelines. However, most patients seek not only for a longer life but also for a life that is worth living (according to their own definition). This problem has been conceptualized as the oncofunctional balance^8–11^, and subspecialized neurosurgeons must face the challenge of optimizing this oncofunctional balance. Whereas noninvasive preoperative functional imaging tools (functional and structural MRI, magnetoencephalography, transcranial magnetic stimulation) are helpful in the first approach of individualized functional mapping (especially in regard to determining language lateralization^12^), the best methodology for functional preservation is to awake the patient and perform continuous intraoperative mapping of cognitive tasks through the use of direct electrical stimulation (DES)^13^. The efficiency of this method has been demonstrated for motor and speech functions^14^. Despite the awareness that functions hosted by the right hemisphere are as important as those hosted by the left hemisphere^15–17^, there are only a few teams opting for awake surgery in right-sided tumors, especially for tumors located in the frontal lobes. One possible explanation would be that there is no study in the literature providing a comprehensive overview of the long-lasting neuropsychological deficits that can be observed after resection of glioma located in the right frontal lobe. Indeed, previous reports in this field were often focused on a single task/function and were somehow neuroscience-oriented^18–20^. As proposed recently, the introduction of a new intraoperative task in awake surgery should be grounded on studies demonstrating that patients operated on without this monitoring do indeed experience debilitating long-lasting neuropsychological deficits^21^. The goal of the present paper is thus to contribute to our knowledge about the frequency and nature of the neuropsychological risks when operating IDH-mutated glioma in the right frontal lobe.

## Methods

### Inclusion criteria

We retrospectively reviewed our consecutive database of cases operated on in awake condition since 2011. We selected all cases with an IDH-mutated glioma located in the right frontal lobe. Clinical and radiological data were retrieved through electronic medical files and the Picture and Archiving Communication System (PACS), respectively.

### Operative techniques

Monitored anesthesia care, which consists of sedation while preserving spontaneous ventilation without any airway instrumentation, was used during the nonawake periods^22^. Sedation was achieved by a mixture of propofol and remifentanil, with additional use of dexmedetomidine in the last cases. Patients were prepared through a systematic protocol that includes hypnotic techniques^23,24^.

All cases were operated on by the senior author, with the naked eye (cases 1–8) or surgical loops (cases 10–20). Surgical microscope was used for case 9. Electrical stimulation was used as previously reported^25–27^. Monitoring was performed by a speech therapist (MB, IP, SL, CPT) and, on surgeon’s request, assessed motor functions (continuous repetitive movement of left superior limb), counting, picture object naming, nonverbal semantic association, and the test “read the mind in the eyes”. Resection was stopped when a functional boundary was encountered.

### Imaging

All patients underwent the same imaging protocol, as previously described^25,27,28^. In this study, the extent of resection was estimated on FLAIR sequences and computed as 100 × (1 − residual volume/initial volume). Surgical cavities were segmented with MI-Brain 2020.04.09 software^29^ (Sherbrooke, Canada, https://github.com/imeka/mi-brain) on 3D-T1 images and resized to a resolution of 1 × 1 × 1 mm^3^. Images were then registered to the MNI template using the *Antsregistration* algorithm and displayed with MRIcro-GL 1.2.20201102 software^30^ (https://www.nitrc.org/projects/mricrogl/). In cases 8, 11, 13–20, language fMRI was performed to confirm the left lateralization of language networks, following the same methodology as previously reported^12^.

### Neuropsychological testing

Patients were thoroughly evaluated neuropsychologically by a speech therapist (MB, IP, SL, CPT) just before, immediately after, and four months after the surgery. After a short non-structured interview with the patient, aiming to record spontaneous complaints, the evaluation assessed language, memory, executive and visuospatial functions, and social cognition. The most common tests were administered to all patients, whereas some tests were added in a patient-specific approach, as expected for evaluations performed in a clinical rather than research context.

Language and semantic cognition testing included:

- DO 80 picture naming^31^,
- Complex language functions including word definitions, word evocation on definition, concatenation of sentences, synonym evocation, antonym evocation and odd word out selection from the TLE^32^ and some parts of the BDAE^33^,
- Writing and reading from the ECLA^34^,
- Understanding of implicit metaphors from the MEC^35^,
- Categorical and literal fluencies (2 min)^36^,
- Nonverbal semantic association (pyramid and palm tree test—PPTT—^37^, or BEC-S in the very last patients^38^.

Tasks tapping attention and executive functions comprised:

- Forward and backward digit span (testing working memory)^39^,
- Paced Auditory Serial Addition Test (PASAT) (testing working memory and sustained attention)^40^,
- Trail-making test, part A & B (testing mental flexibility)^36^,
- Stroop test (testing inhibition)^41^,
- d2-attention test (testing sustained attention)^42^,
- Copy of the Rey figure (testing visuospatial praxies)^43^.

Visuospatial cognition was assessed by line bisection, Bells’ and Clock’s tests and writing^44^. Memory was evaluated through delayed copy of the Rey figure and RI-RL 16 task^45^ (or RI 48 in the very first patients^46^). Finally, social cognition was evaluated with the Read the Mind in the Eyes test^47^, facial emotion recognition^48,49^, and faux pas recognition^49,50^.

For each patient, speech therapists wrote a synthetic conclusion summarizing the patient’s performance in terms of nosological entities (deficits of executive functions, attention disorder, short-term memory impairment, etc.). In the results section, we listed for each patient and for each evaluation the key words retrieved from these conclusions. We claim that this approach allows us to obtain a picture of patients’ functions that is easier to grasp and interpret than the full set of raw psychometric scores. The main scores and their corresponding z-scores are nonetheless also given at the group level. Moreover, z-scores were used to categorize each patient as having a long-term impairment in one domain when at least one test z-score of that domain decreased by 1.5 units or more.

### Statistical methods

Differences between pre and postop scores were assessed using paired test. Normality of these differences was checked using Shapiro test. For normal data, we used a paired Student’s t-test. For non-normal data, we used a paired Wilcoxon test. Significance was set at a *p* value of 0.05. All analysis were performed with R^51^ under R studio software^52^.

### Ethical approval

All procedures performed in studies involving human participants were in accordance with the ethical standards of the institutional research committee of Lariboisière Hospital and with the 1964 Helsinki declaration and its later amendments. The study was approved by the local ethics committee Pôle Neurosciences of Lariboisière hospital. Informed consent was obtained from all individual participants included in the study.

## Results

### Patients characteristics

Twenty surgical cases in eighteen patients (two patients operated on twice) were included in the study. There were 6 females and 12 males. Among the 20 cases, 5 were redo surgeries. Symptoms motivating the first MRI were generalized seizures in 13 out of 18 patients and persisting headaches in one patient. Radiological discovery was incidental in 4 patients. Median age at surgery was 42.5 years (range 26–58). All patients were right-handed, except one patient (case 5) who was ambidextrous. Left lateralization of language networks was confirmed in the 10 patients in whom fMRI was performed. Patients were working at the time of their surgery in seventeen out of twenty cases. Four patients received an adjuvant treatment (two chemotherapy and two concomitant chemo-radiotherapy) within the four months interval between surgery and neuropsychological evaluation. Patients’ characteristics are summarized in Table 1.

**Table 1:** Patients and tumors characteristics. *GS* generalized seizure, *ID* incidental discovery, *RH* right-handed, *Amb* ambidextrous, *MFG* middle frontal gyrus, *SFG* superior frontal gyrus, *IFG* inferior frontal gyrus, *OII* grade II oligodendroglioma, *AII* grade II astrocytoma, *OIII* grade III oligodendroglioma, *GBM* glioblastoma, *NA* not applicable.

	Age	Sex	Initial symptom	Manual dominance	Redo	Location	Initial volume (cc)	Residual volume (cc)	EOR (%)	Histo	Postop therapy	Long-lasting cognitive impairment	Working at diagnosis	Work resumption	Follow-up
1	58	M	ID	RH		MFG	13.5	0.25	98	OII	No	Inhibition, flexibility, speed processing	Yes	No	102
2	41	F	GS	RH		SFG	63	8.4	87	AII	No	0	Yes	Yes	90
3	44	M	GS	RH	Yes	MFG + antero-basal	30	0	100	AII	No	Flexibility	Yes	Yes	78
4	37	F	GS	RH		SFG	33	0	100	AII	No	Speed processing	Yes	Yes	60
5	28	F	GS	Amb		Basal	62.4	0.6	99	OIII	No	word finding	No	NA	58
6	47	M	ID	RH		IFG + insula	42	0	100	OII	No	spatial cognition, flexibility, speed processing, short-term memory, social cognition	Yes	Yes	56
7	38	M	GS	RH		SFG + MFG + IFG	175	40	77	AII	No	speed processing	Yes	No	48
8	40	M	ID	RH		MFG	13	0	100	OII	No	0	Yes	Yes	42
9	51	M	GS	RH	Yes	Antero-mesial	21	1	95	OIII	No	0	Yes	Yes	42
10	39	M	GS	RH		SFG + MFG	134	19	86	AII	No	0	Yes	No	36
11	26	M	GS	RH		SFG	79	6	92	GBM	CT-RT	0	No	NA	34
12	32	M	Headaches	RH		Antero-mesial	103	0	100	OIII	CT-RT	Flexibility	Yes	Yes	30
13	49	M	NA	RH	Yes	IFG + insula	5.7	0	100	OIII	No	0	Yes	Yes	30
14	45	M	GS	RH		SFG	32.5	0	100	AII	No	0	Yes	No	24
15	54	F	GS	RH		SFG	30	0	100	AII	No	Attention, flexibility, spatial cognition	Yes	No	18
16	47	F	NA	RH	Yes	SFG + MFG	16	10	38	GBM	CT	NA	No	NA	18
17	31	M	ID	RH	Yes	Antero-mesial	1.7	0	100	AII	No	0	Yes	Yes	18
18	54	M	GS	RH		SFG	41	0	100	AII	No	0	Yes	Yes	18
19	32	F	GS	RH		Antero-basal	48	0	100	OII	No	0	Yes	Yes	12
20	45	F	GS	RH		SFG + MFG + IFG	83.1	12.5	85	AII	CT	Metaphoric language, social cognition	Yes	Yes	12

### Tumor characteristics

The median preoperative volume was 37 cc (mean 51 cc, range 1.7–175 cc). Preferential locations were the posterior part of the superior frontal gyrus (SFG), followed by the anterior frontal lobe, the middle frontal gyrus (MFG), and inferior frontal gyrus (IFG). Contrast enhancement was present in 4 cases. Histopathological examination revealed a grade II in 70% of cases (1/3 of 1p-19q co-deleted oligodendroglioma, 2/3 of astrocytoma), a grade III in 20% of cases (all 1p-19q co-deleted oligodendroglioma) and a glioblastoma in 10% of cases. Tumor characteristics are summarized in Table 1.

### Surgical results

**Figure 1:**
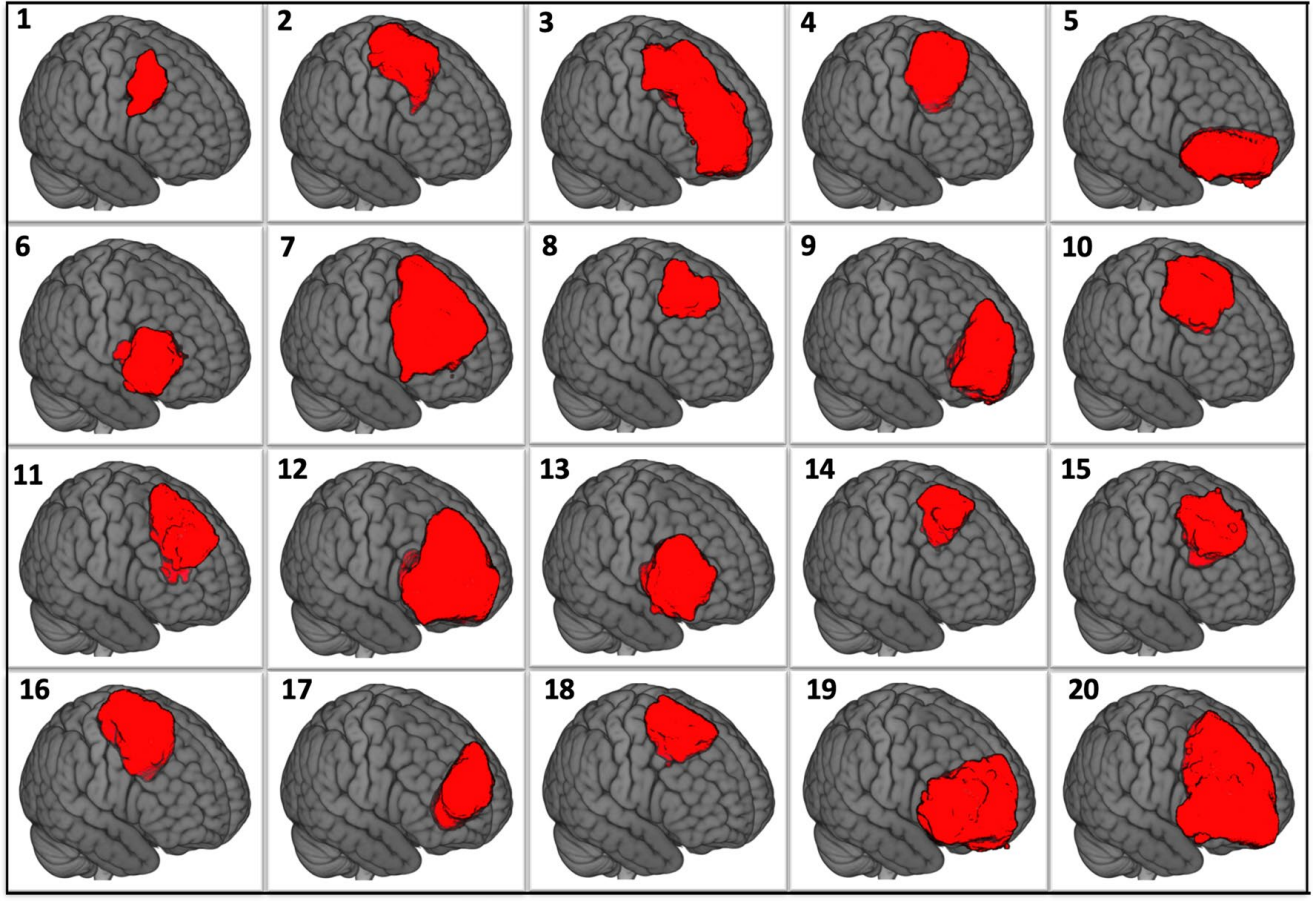
Surgical cavities for the 20 cases after registration in the MNI template.

The mean extent of resection was 93% (range 37.5–100%), and the mean residual volume was 4.9 cc (range 0–40 cc). Resections were complete in 55% of surgeries. Figure 1 shows the surgical cavities after registration to the MNI template.

None of the patients presented long-lasting postoperative motor deficits. Two patients presented incomplete akinesia, which resolved within a couple of days. This akinesia affected both the upper and lower extremities (case 2) or only the upper extremity (case 10). One patient (case 1) had an epidural hematoma requiring evacuation at postoperative day 3. One patient (case 15) had a wound infection requiring bone flap removal 3 months after the surgery and a cranioplasty 6 months later.

### Mapping results

**Figure 2:**
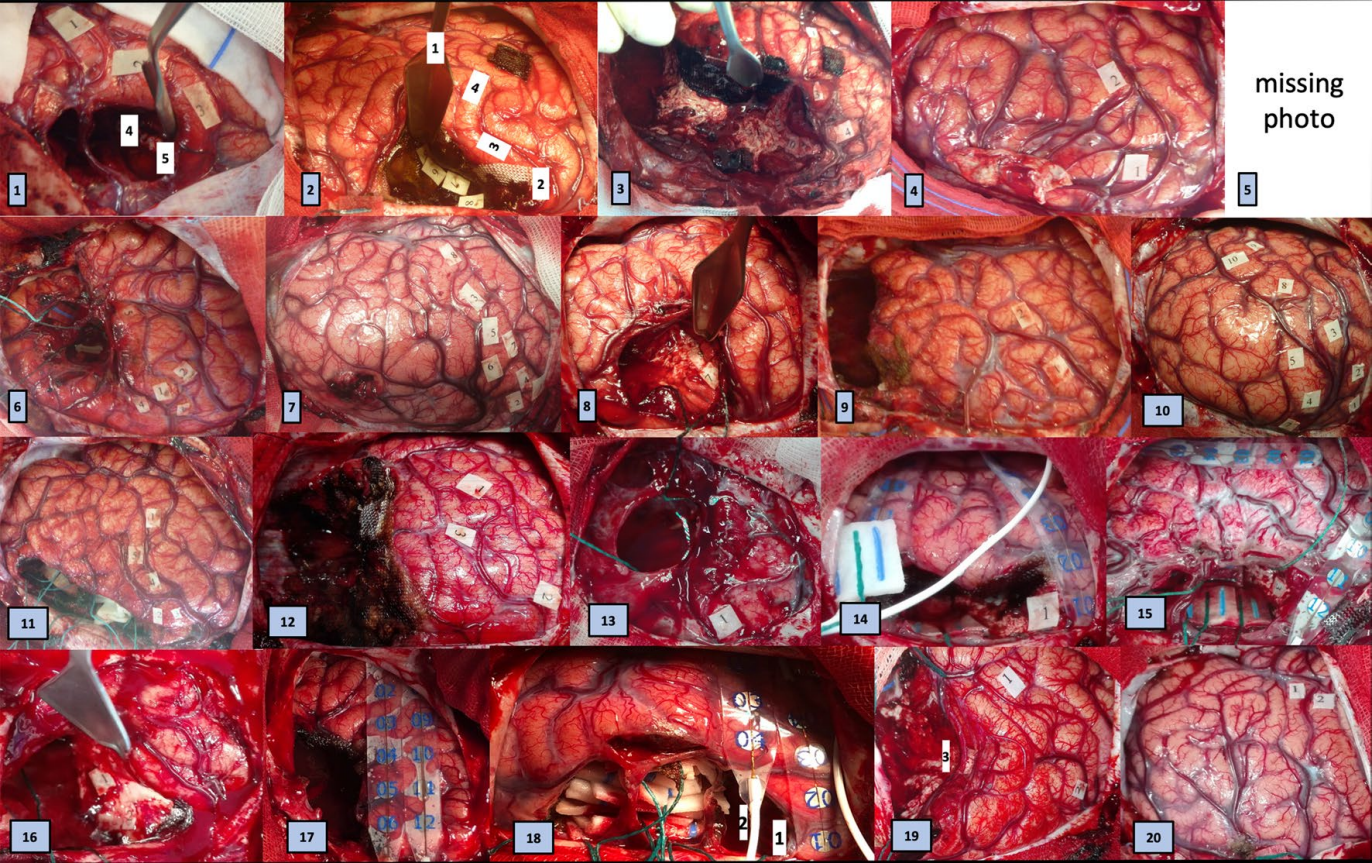
Photographies of intraoperative functional mappings for the 20 cases. No photography was found for case 5.

All mapping results are given in Table 2 and Fig. 2. For all 19 patients in whom the precentral gyrus was exposed, stimulation generated positive motor responses. Sites generating motor arrest (of speech and/or of upper limb movement) were seen in 12 cases. No reproducible cortical sites were found when monitoring nonverbal semantic association (PPTT) or emotion recognition (RME). When stimulating the white matter, positive motor responses were seen in 5 cases (upper limb on 1 site, lower limb in 5 sites). White matter sites of upper extremity motor arrest were observed in 12 cases. Eye movements with loss of contact were noted in 3 cases. No reproducible sites were found when testing the PPTT or RME. Finally, stimulation of white matter generated in two patients (cases 17 and 19) made it impossible to perform the 1-back naming task combined with continuous repetitive movement of the upper extremity. In both cases, patients spontaneously reported an attentional disorder: one patient said ‘I do not know, I did not pay attention’, and the other said ‘I do not know, I did not see the last image’.

**Table 2:** Functional sites intraoperatively identified. The numbers in brackets refer to the tag number on respective photographies of Fig. 2. MA = motor arrest ; LE = lower extremity ; UE = upper extremity.

	Tasks	Intensity	Cortical sites	Tasks	Intensity	White matter sites
1	Rest	1.5 mA	Tongue movement (2) Chin movement (3) Tongue tingling			
	Counting while moving UE	1.5 mA	Speech MA (1)	Naming while moving UE	2 mA	Slowing (4) Complete MA (5)
2	Rest	1 mA	Face movement (1,4) UE movement (2,3)	Rest (intraop akinesia)	3 mA	UE movement (5,6,7) Leg movement (8)
	Counting while moving UE	2 mA	Ø			
3	Rest	1.5 – 2 mA	Mouth movement (1) Jaw movement (2) Thomb-index pinch (4)			
	Counting while moving UE	2 mA	Complete MA (3)	Naming while moving UE	2 mA	Complete MA + FEF (10,11,12)
4	Rest	1 mA	Face movement (2) Hand movement (1)			
	Counting while moving UE			Naming while moving UE	2 mA	UE MA + leg movement ( NA) Leg movement (NA)
5	Rest	1 mA	Face movement (NA)			
	Counting while moving UE	2 mA	Ø	Naming while moving UE	2 mA	Ø
	Naming					
	PPTT					
	RME					
6	Rest	1.5 mA	Tongue MA (1,4) Tongue tingling (2) Wrist extension (3)	Rest	2 mA	Ø
	Counting while moving UE	2 mA	Complete MA (5)	Naming while moving UE		UE MA (NA)
	PPTT		Ø			
	RME					
	Numbers bisection					
7	Rest	1 mA	Wrist movement (1,2) Face movement (3) Fingers flexion (4)			
	Counting while moving UE		Complete MA (5,6,7,8)	Moving LE	2 mA	Foot contraction Foot MA (NA)
	Naming while moving UE	2 mA	Ø	Naming while moving UE or LE	3 mA	Acceleration Deceleration Foot contraction (NA) Complete MA (NA)
	PPTT			PPTT		Ø
8	Rest	1 mA	Thumb tingling (2)			
	Counting while moving UE	1–2 mA	Dysarthria and slowness of speech (1) UE MA (4) Speech arrest (3) Complete MA (5) Eye movement & loss of contact (6)	Bilateral antiphasic movement of UEs	2 mA	Inhibition of coordination only (7,8)
	PPTT	2.5 mA	Non-reproducible errors in pars triangularis			
9	Rest	1 mA	Thumb and wrist movement (1)			
	Counting while moving UE	1 mA	Complete MA (2)	PPTT namng	2 mA	Ø
	PPTT	2 mA	Non-reproducible errors in pars triangularis and posterior part of MFG	PPTT pointing		
	RME		Ø			
10	Rest	1 mA	Hand tingling (1,2) Face tingling (3) Thumb movement (4) Fingers movement (5) Eyes closing (8)			
	Counting while moving UE	1.5 mA	UE MA (7) Complete MA (10) Dysarthria and slowness of speech (9)	Counting while moving UE	4 mA	Toes and pain in the prostate (NA)
	PPTT	2 mA	Ø			
11	Rest	1 mA	Tongue tingling (1) Wrist movement (2) Fingers flexion (3) Thomb movement (4) Face movement (5)	Rest	2.5 mA	Fingers movement (NA)
				Counting while moving UE	2.5 mA	UE MA (NA)
	PPTT	2 mA	Ø	PPTT	2.5 mA	Ø
12	Counting while moving UE	1 mA 2 mA	Complete MA (1) Eyes movement and loss of contact (2)			
	PPTT	2 mA	Reproducible errors (3)	PPTT	5 mA	Arrest (NA)
13	Counting while moving UE	1 mA	Speech arrest (1)	Counting while moving UE	2 mA	Hand tingling (NA) Eye movement (NA)
				PPTT	2 mA	Ø
				RME		
14	Rest	4 mA	Fingers movement			
	Naming while moving UE	3 mA	Ø	Moving UE	3 mA	UE MA (1)
	Bilateral antiphasic movement of UEs					
	PPTT			PPTT		Non-reproducible errors in the corona radiata
15	Rest	2.75 mA	Thumb movement (2)			
	Counting while moving UE	1.5 mA	Complete MA (1)	Counting while moving UE	2.75 mA	UE MA (3)
	Naming while moving UE	2.75 mA	Ø			
	PPTT					
	RME					
16	Rest	0.75 mA	UE movement (NA)	Rest	2 mA	Leg movement (2)
				Moving UE		UE MA (1)
17	1-back naming while moving UE	2 mA	Ø	1-back naming while moving UE	2 mA	Attention disorder (NA)
18	Rest	1.5 mA	Hand movement (NA) Fingers movement (NA)	Rest	1.5 mA	UE movement (NA)
	Counting while moving UE		UE MA (NA) UE MA with leg movement (NA)	Counting while moving UE		Vocalization with eye movement (NA)
19	Rest	1.5 mA	Ø			
	Counting while moving UE		Complete MA (1) Eye movement and loss of contact (2)			
	1-back naming while moving UE	2 mA	Ø	1-back naming while moving UE	3 mA	Attention disorder (3)
	PPTT			PPTT		Ø
	RME			RME		
20	Rest	1.5 mA	Nausea (in the precentral gyrus)			
	Counting while moving UE		Complete MA (1,2)			
	1-back naming while moving UE	3 mA	Ø	1-back naming while moving UE	3 mA	UE MA (NA)
	PPTT			PPTT		Ø
	RME			RME

### Group-level analysis of neuropsychological quantitative evaluations

Table 3 gives the quantitative means of the raw scores and z-scores for picture naming, Rey figure copy, digit span forward and backward, verbal fluencies, Trail Making Test (B-A), Stroop (conflict), and Bells’ test. Preoperatively, all z-score means were in the normal range (> − 1.0), in accordance with almost normal cognitive functioning in IDH-mutated glioma patients. In the immediate postoperative period, a statistically significant (*p* < 0.05) deterioration was observed for DO80, Rey figure, verbal fluencies, TMT B-A, Stroop test and Bells’ test. At the late postoperative evaluation, only categorical fluency significantly differed from its preoperative value (mean 33.1 postop vs 36.3 preop).

**Table 3:** Group-level analysis of cognitive performances. Raw scores and z-scores of the main cognitive tasks are given at preoperative, immediate postoperative and late postoperative evaluations. The values in bold are considered as pathologic (z-scores < − 1.5), while values with a star differed significantly (*p* < 0.05) from their preoperative values.

	Preoperative performance				Immediate postop performance				Late postop performa			
	Mean	Median	Max	Min	Mean	Median	Max	Min	Mean	Median	Max	Min
DO80	77.8	79.0	80.0	58.0	74.9*	78.0	80.0	43.0	77.8	80.0	80.0	54.0
DO80 (z-score)	− 0.9	0.1	0.8	− 14.7	− 3.3*	− 0.9	0.7	− 28.0	− 0.9	0.7	0.8	− 17.5
Rey Figure	34.6	36.0	36.0	26.5	33.4*	34.5	36.0	19.0	35.0	36.0	36.0	31.0
Rey Figure (z-score)	− 0.3	0.4	0.8	− 5.1	− 1.1*	− 0.4	0.8	− 8.9	− 0.1	0.4	0.8	− 3.4
Rey Figure time (s)	158.3	137.0	319.0	56.0	215.4	147.0	515.0	93.2	105.2	104.0	179.0	45.0
Rey Figure time (z-score)	0.9	1.3	2.0	− 1.1	0.3	0.8	1.8	− 3.7	1.4	1.4	2.5	0.4
Span forward	5.8	6.0	8.0	4.0	5.4	5.0	8.0	3.0	5.4	5.0	7.0	4.0
Span forward (z-score)	− 0.5	− 0.5	1.8	− 3.1	− 0.9	− 0.9	1.0	− 2.5	− 0.9	− 0.7	0.7	− 3.1
Span backward	4.2	4.0	6.0	3.0	3.8	3.5	6.0	2.0	4.1	4.0	7.0	2.0
Span backward (z-score)	− 0.5	− 0.7	1.7	− 1.4	− 0.8	− 1.1	1.7	− 2.1	− 0.5	− 0.7	2.9	− 2.1
Rey Figure recall	23.6	24.0	35.0	13.0	22.4	24.3	32.0	6.5	26.0	26.0	34.0	16.0
Rey Figure recall (z-score)	0.0	0.2	1.8	− 1.4	− 0.3	0.2	1.2	− 3.6	0.4	0.5	1.6	− 0.9
Categorical fluency	36.3	36.0	55.0	18.0	24.2*	22.0	34.0	12.0	33.1*	32.0	45.0	22.0
Categorical fluency (z-score)	0.3	0.4	2.1	− 1.9	− 1.1*	− 1.4	1.4	− 3.0	− 0.1*	− 0.2	1.3	− 1.2
Literal fluency	25.2	25.0	45.0	14.0	18.0*	17.0	33.0	8.0	24.4	23.0	38.0	14.0
Literal fluency (z-score)	0.2	0.0	3.2	− 1.4	− 0.9*	− 1.1	1.2	− 2.7	0.1	− 0.1	2.1	− 1.2
TMT B-A (s)	39.3	28.0	107.0	1.0	**71.2**	40.0	366.0	− 81.0	52.9	42.0	171.0	14.0
TMT B-A (z-score)	0.0	0.3	1.7	− 3.3	**− 1.8**	− 0.5	0.9	− 7.2	− 0.5	0.1	1.1	− 3.6
Stroop conflict (s)	111.2	105.0	258.0	66.0	154.1*	116.0	461.0	79.0	111.1	104.0	298.0	65.0
Stroop conflict (z-score)	− 0.2	− 0.1	1.5	− 5.1	− 1.8*	− 0.8	0.9	− 11.6	− 0.3	− 0.1	1.5	− 6.4
Bell	34.1	34.0	35.0	30.0	29.1*	31.0	35.0	12.0	34.1	34.0	35.0	32.0
Bell (z-score)	0.5	0.5	1.0	− 1.4	− 1.8*	− 0.9	1.0	− 9.7	0.5	0.5	1.0	− 0.4
Bell time (s)	128.0	121.1	274.0	52.0	191.8*	195.0	472.0	73.9	124.2	117.0	326.0	64.1
Bell time (z-score)	− 0.5	− 0.2	1.3	− 4.0	− 2.0*	− 2.1	0.7	− 8.6	− 0.4	− 0.1	1.0	− 5.2

### Individual-level analysis of preoperative neuropsychological evaluation

Preoperatively, patients rarely reported spontaneous cognitive or behavioral disorders. (see Table 4). The most common complaints were distractibility (30% of cases), followed by fatigability (20%) and irritability (15%). Neuropsychological evaluations demonstrated mild deficits (see Table 5). These deficits impacted executive functions in 45% of cases, attention in 45% of cases, and verbal short-term memory in 45% of cases. Speed processing was also slightly below the average in 50% of cases. Of note, difficulties with high-level semantic cognition (conceptualizing or grasping implicit) were observed in 20% of cases.

**Table 4:** Subjective complaints spontaneously reported by patients preoperatively and 4 months after surgery.

	Preoperative evaluation		Postoperative 4 months evaluation	
	Rate (%)	Cases (#)	Rate (%)	Patient-cases (#)
Fatigability	20	7, 10, 11, 20	65	1, 2, 3, 4, 5, 7, 9, 11, 14, 17, 18, 19, 20
Distractibility	30	2, 4, 10, 11, 16, 20	45	1, 2, 3, 4, 5, 6, 7, 9, 14
Multitasking	0		30	1, 2, 4, 5, 7, 19
Processing speed	0		15	4, 19, 20
Lack of motivation/apathy	0		15	5, 7, 17
Difficulties related to time	0		15	1, 3, 7
Time perception	0		5	3
Schedule management	0		10	1, 7
Urinary urgency	0		10	10, 14
Irritability	15	6, 16, 20	10	2, 7
Mood disorder	0		10	4, 14
Loss of bimanual coordination	0		5	2
Language disorder	5	5	5	5
Sleep disorder	0		5	14

**Table 5:** Objective neuropsychological evaluations.

	Preoperative evaluation		Immediate postoperative evaluation		4 months postop evaluation	
	Rate (%)	Patient-cases (#)	Rate (%)	Patient-cases (#)	Rate (%)	Patient-cases (#)
Executive functions	45	1, 3, 5, 7, 8, 11, 13, 15, 20	75	1, 2, 3, 5, 6, 8, 9, 10, 11, 14, 15, 16, 17, 18, 20	65	1, 2, 3, 4, 5, 6, 7, 8, 11, 12, 15
Flexibility	30	3, 5, 7, 8, 11, 13	65	1, 2, 3, 5, 6, 8, 9, 11, 14, 15, 16, 18, 20	40	1, 3, 4, 5, 6, 8, 12, 15
Planning	5	20	NA		20	1, 2, 4, 7
Inhibition	20	1, 5, 8, 15	30	1, 2, 8, 9, 10, 17	15	1, 8, 15
Updating	0		5	20	10	8, 11
Attention	45	1, 2, 3, 6, 8, 11, 12, 13, 20	65	1, 2, 3, 5, 6, 7, 8, 9, 11, 12, 17, 18, 20	45	1, 2, 3, 5, 6, 7, 8, 15, 20
Short-term memory	45	1, 3, 4, 5, 11, 13, 17, 19, 20	25	1, 6, 15, 16, 19	35	1, 2, 3, 4, 6, 15, 20
Spatial cognition	0		60	1, 2, 3, 6, 7, 8, 10, 11, 12, 14, 15, 16, 18, 20	10	6, 15
Speed processing	50	2, 3, 5, 7, 11, 12, 14, 15, 16, 20	65	3, 5, 6, 7, 8, 11, 13, 14, 15, 16, 17, 19, 20	25	1, 5, 6, 7, 15
Social cognition	5	8	15	6, 8, 20	15	6, 11, 20
Anosodiaphoria	0		20	1, 6, 15, 20	15	5, 6, 20
Anosognosia	0		25	1, 3, 6, 12, 16	0	
Apathy	0		30	2, 7, 8, 11, 12, 19	0	
Aprosodia/Amimia	0		45	2, 3, 7, 8, 11, 12, 16, 19, 20	0	
Emotional sensitivity	0		10	2, 11	0	
Low-level semantics	5	5	15	5, 6, 8	15	5, 8, 12
High-level semantics	20	5, 13, 14, 20	20	1, 6, 14, 20	15	6, 15, 20
Implicit	10	13, 14	10	1, 6, 14	10	6, 15
Metaphores	0		5	20	5	20
Conceptualization	10	5, 20	5	20	0	
Haste	10	7, 13	0		10	6, 19
Fatigability	5	13	5	9	5	3

### Individual-level analysis of immediate postoperative evaluation

At the immediate (within one week postsurgery) postoperative evaluation, 75% of cases had marked deficits in executive functions (see Table 5). Attention capabilities were also strongly impacted in 65% of cases. Left unilateral spatial neglect (USN) was detected in 60% of cases. Behavioral disturbances included apathy (30% of cases), aprosodia/amimia (45% of cases), and emotional sensitivity (10% of cases). Of note, anosognosia was observed in 25% of cases.

### Individual-level analysis of postoperative neuropsychological evaluation

All but 4 patient cases underwent intensive cognitive rehabilitation for a period of four months. Patients performed this cognitive training in the outpatient speech therapy clinics nearest to their home.

At 4 months postsurgery, the complaints most commonly reported by patients were fatigability (65% of cases), distractibility (45% of cases) and difficulties coping with multitasking (30% of cases) (see Table 4). Uncommon complaints included reduced speed processing, lack of motivation, difficulties with time (either for time perception or for schedule management), urinary urgency, irritability, mood disorder, loss of bimanual coordination, language disorder and sleep disorder. Objective neuropsychological evaluations confirmed these self-reported lamentations (see Table 5). Executive abilities and attention were the main affected functions, together with verbal short-term memory. Interestingly, signs of USN almost completely resolved (two patient cases with very mild persisting signs of left USN). Importantly, a small proportion of patients had persistent disorders of high-level semantic cognition (grasping implicit or metaphors) and/or an impairment of social cognition. Overall, when comparing the pre- and postoperative evaluations, 9 out of 20 cases demonstrated decreased performance in at least one domain among executive functions, speed processing, attention, spatial cognition, semantic cognition, and social cognition.

### Clinical follow-up

Out of the seventeen patients working at the time of surgery, twelve (70%) resumed their professional activity within 6 months after the surgery. All patients but one were alive at the time of last follow-up: one patient (case 10) died after 3 years of glioma evolution. Median follow-up was 42 months (range 12–102 months).

## Discussion

To the best of our knowledge, our study is the first to provide a comprehensive overview of the cognitive dysfunctions that might remain four months after awake resection of IDH-mutated glioma located to the right frontal lobe. Such knowledge can help neurosurgeons better inform their patients about the (mild) cognitive risks that come with resection of a right frontal IDH-mutated glioma. We would like to put our results in perspective with the previous literature*.*

### Motor control

In the present series, only two patients experienced transient akinesia, which is typical of SMA syndrome. For both of them, akinesia occurred intraoperatively before sites of motor arrest could be properly identified. In all other patients, such sites were detected and preserved, thus avoiding transient postoperative akinesia, as previously reported^53^. It should be emphasized that it is now recognized that the recovery of SMA syndrome is incomplete and that disorders of fine motor movements might persist, in particular regarding bimanual coordination, a subjective complaint reported by one patient (case 2). Of note, two patients also reported urge incontinence, as previously observed^54^. These symptoms hampered their quality of life but improved under 5 mg solifenacine succinate twice daily.

### Neuropsychological outcomes: group-level analysis

Our group-level analysis could not capture the mild long-term deficits encountered in this selected group of patients (except for a slight decrease in categorical fluency). Although we cannot rule out that this is due to the small size of the series, such a result is in good accordance with the high level of recovery observed in this patient population (thanks to the efficient implementation of plasticity mechanisms)^55^. It can be hypothesized that such favorable cognitive outcomes—in spite of a large extent of resection—were achieved thanks to intraoperative mapping relying on tasks tapping cognitive control abilities. As an alternative hypothesis, averaging at the group level might have balanced improved and deteriorated patients’ scores. Hence, we next investigated evaluations at the individual level by analyzing patients’ self-reported complaints, quantitative changes in psychometric z-scores, and objective qualitative conclusions found in the written reports of the speech therapists.

### Neuropsychological outcomes: individual-level analysis

Very few studies have preoperatively explored the cognitive functioning of patients with right frontal glioma, and even fewer have reported subjective complaints, as explained by the patients themselves. Eight out of the fifteen patients with an incidental glioma studied by Cochereau et al.^56^ had a tumor located in the right frontal lobe. Five out of the eight had subjective complaints, including tiredness, altered attention, and irritability. Our results are perfectly in line with this study, as fatigability, distractibility and irritability were reported by 20, 30 and 15% of patients in our series, respectively (see Table 4). Objective evaluations demonstrated deficits in working memory and/or executive functions in four out of the eight patients reported by Cochereau et al. Similarly, we found that executive functions, short-term working memory, and attention were the most commonly impacted domains, with almost half of the patients being affected (see Table 5). It is worth emphasizing that these deficits were very mild, in accordance with the high rate of patients with professional activity just before the surgery (17 out of 20 patient cases). Interestingly, impairments of high-level semantic cognition (grasping metaphors or implicit) were diagnosed in 20% of cases. Such troubles have been previously reported after resection of right hemispheric glioma^57^ and deserve further specific investigations. Of note, we found a low rate of preoperative disturbance in social cognition, which is also in line with a recent report^20^.

While almost every patient presented cognitive deterioration at the immediate postoperative evaluations, slight impairment in at least one domain (among executive functions, attention, speed processing, spatial cognition, semantic cognition, or social cognition) was detected at the four-month evaluation in only 9 cases out of 20. Nonetheless, the decline was slight enough that a remarkably high proportion (70%) of patients working preoperatively could resume their work within six months after the surgery. Again, this good outcome suggests that awake cognitive mapping could have contributed to preserving the patients’ socioprofessional life.

Our results are in line with a previous study^58^ reporting a decline in executive functions and/or speed processing and/or attention in 32% of cases (both left and right hemispheres). Resection map symptom mapping highlighted the right frontal lobe as being the location most at risk^58^. Such results were further confirmed by studies in 77 low-grade glioma patients, including 27 cases of right frontal location^59^: preoperative impairments in verbal memory, finger tapping, symbol digit coding, cognitive flexibility, verbal fluency and sustained attention were observed, with further deterioration at three months for sustained attention. Two other recent studies also emphasized the risk regarding inhibition capabilities (as measured by Stroop’s task) when operating in the right frontal lobe^19,60^. Regarding visuospatial cognition, long-lasting left USN was found in one-third of patients in whom resection of right hemispheric tumors encompassed the SFG and MFG^18^. In our series, whereas USN was found in 60% of cases in the immediate postoperative period, mild signs of USN were found in only 10% of cases four months later (in particular, none of the patients deviated at the line bisection task). To explain the difference between the two series, it is tempting to put forward the following hypothesis, already mentioned in^18^: persistent deficits would be caused by the cumulative effect of resecting both the first and second branches of the superior longitudinal fasciculus, a situation that might have been less frequent in the present series.

Performances in social cognition declined in two patients, in accordance with a previous report^61^. It should be noted that we failed to identify reproducible stimulation sites disturbing the RME task, contrary to previous reports^20,62^. The lack of experience of the team regarding this kind of mapping likely explains this difference. An alternative explanation could be that the stimulated area is too small compared to the cortical area supporting the function. This latter hypothesis could be tested by simultaneously stimulating two sites, as recently suggested^63^. Similarly, we found no reproducible sites when testing the nonverbal semantic association task (PPTT), contrary to previous reports^64,65^. It can be hypothesized that the identification of such sites would have contributed to preventing the postoperative semantic cognition disorders (implicit and/or metaphors understanding) found in three cases.

Finally, objective evaluations and subjective complaints overlapped only partially. Some dysfunctions reported by patients were indeed not captured by the battery of tasks we used. Such functions include fatigability, irritability, or multitasking. Specific tasks should be designed to objectify and quantify these kinds of impairments.

### Limitations

Finally, our study has several limitations, including all those that come with a retrospective design and a small sample size, making it difficult to generalize definitive conclusions. However, the fact that cases were consecutively reported and that the management was the same for all patients partly compensated for these limitations. Mixing histological grade might have blurred the results, as grade might interfere with neuroplasticity capabilities. However, as we selected only IDH-mutated glioma, this variability was strongly reduced. Indeed, in our subgroup of patients—and even in case of grade IV-, the tumor remained under control for several months after surgery and postoperative adjuvant treatment, hence providing a large time window for plasticity implementation. The cognitive evaluations were performed by four different speech therapists, and this might have introduced heterogeneity in the qualitative reports, but this is compensated by the extensive quantitative data of our test battery. Furthermore, patients were evaluated only at 4 months, so we cannot rule out that a different pattern of deficits would have been seen one year later. However, there are some data in the literature demonstrating that, in general, the cognitive recovery curve reaches a plateau after 4 months (see, for example^66^, for spatial attention and awareness). Hence, although this is not proven, we made the reasonable assumption that the 4-month measure is a good proxy of the 1-year measure. Last but not least, the small size of our series did not allow us to perform a multivariate analysis that would have included all regressors known to influence cognitive recovery, including age, preoperative cognitive status, somatic gene polymorphisms^67^, location and extent of resection, and growth rate of residual tumor. We thus emphasize the need to share data between centers to address such important questions.

## Conclusion

Overall, the present study supports the idea that the right frontal lobe should be considered a highly eloquent area, given the high rate of persistent mild neuropsychological impairments found 4 months after surgery. There is still much to do to better understand the neuronal networks sustaining these high-level functions and, most importantly, to better understand how resection will impact those networks, in particular for differentiating damages that will be restorable through plasticity-mediated reorganization from those that will overwhelm the potentialities of plasticity and cause definitive deficits. This is a real challenge, considering the high degree of individual variability of topographical organization and plasticity of cognitive networks and meta-networks^68–71^. Finally, the encouraging high rate of work resumption gives support to the assumption that awake surgery could have a positive impact on the patients’ socioprofessional life: intraoperative monitoring of executive functions, semantic cognition and social cognition in an awake patient might be currently the best method to preserve these functions, thus giving to each individual patient the best chances to return to a normal socioprofessional life. Such an assumption deserves confirmation from future studies with larger samples.
